# Standards for Cell Line Authentication and Beyond

**DOI:** 10.1371/journal.pbio.1002476

**Published:** 2016-06-14

**Authors:** Jamie L. Almeida, Kenneth D. Cole, Anne L. Plant

**Affiliations:** Biosystems and Biomaterials Division, The National Institute of Standards and Technology, Gaithersburg, Maryland, United States of America

## Abstract

Different genomic technologies have been applied to cell line authentication, but only one method (short tandem repeat [STR] profiling) has been the subject of a comprehensive and definitive standard (ASN-0002). Here we discuss the power of this document and why standards such as this are so critical for establishing the consensus technical criteria and practices that can enable progress in the fields of research that use cell lines. We also examine other methods that could be used for authentication and discuss how a combination of methods could be used in a holistic fashion to assess various critical aspects of the quality of cell lines.

## Abstract 2

While definitive methods for human cell line authentication have been compiled in a documentary standard, this Perspective maintains that more research is required to provide similar vetting of methods for non-human cell lines.

## A Comprehensive Approach to Assessing the Quality of Human and Nonhuman Cell Lines

Cell line authentication is still poorly reported, despite the prevalence of misidentification, the calls for community action, and the estimates of wasted research dollars [1–6]. Studies that are carried out with misidentified cell lines add misinformation to the literature, are likely not to be reproducible, and can spur additional studies that are also of questionable value [7]. The general reluctance by research labs to perform and report results that establish the identity and purity of their cell lines is one of the contributing factors to irreproducibility of biomedical research results [8,9]. Cell line authentication is an example of the kind of data that add confidence to the results of a scientific study. The lack of reporting of cell line authentication data reflects a broader failure to appreciate the need for more complete reporting of experimental details that qualify data and provide confidence in research results [10]. Without sufficient control data that provide assurance that the study results are based on reasonable assumptions, the value of the data is questionable. To establish a high level of confidence that published data are reliable and can be confidently built on by others, data that support assumptions about reagents, instrument performance, assay validity, software algorithm accuracy, and cell lines are critical [10]. One might argue that these control data are as important as the study data themselves.

The critical nature of the issue of cell line authentication has led to requirements being put in place by funding agencies and publishers for authentication and purity of cell lines. The National Institutes of Health (NIH) has revised guidelines to applications for funding [11] and provided guidelines for reporting that many journals (which are listed in the announcement) have endorsed [12]. Since 2013, the Nature publishing group has required authors to report the status of the authentication of cell lines used. A web site (http://www.scoop.it/t/cell-line-contamination/p/4040895974/2015/04/08/which-journals-ask-for-cell-line-authentication) lists journals with authentication policies and links to those policies. The Prostate Cancer Foundation has had an initiative requiring cell line authentication and contamination testing for grantees since 2013, and since then a campaign to encourage cell line authentication has been initiated by the Global Biological Standards Institute [13] with cosignatories that include a number of nonprofit research funding organizations. The University of Texas MD Anderson Cancer Center has an institutional policy requiring cell line testing. Workshops on the topic include the NIH Workshop on Reproducibility in Cell Culture Studies [14].

Why are more labs not authenticating their human cell lines? Possibly, many researchers are unaware of the existence of a documentary standard that codifies specific guidance and laboratory protocols and the availability of authentication services that can make the process relatively easy. The American National Standards Institute (ANSI) and the American Type Culture Collection (ATCC) provide a documentary standard (ASN-0002) for human cell lines based on the use of short tandem repeats (STR) entitled *Authentication of Human Cell Lines*: *Standardization of STR Profiling*. This is a very thorough and helpful document that explains why, and precisely how, to authenticate human cell lines [15], and provides a definitive compilation of methods and results from recognized experts.

Unlike the situation for human cell lines, authentication of nonhuman cell lines is less clear and is burdened by the immature state of development of kits, testing services, databases, and accepted standards for STR or other DNA markers.

In addition to authentication, there are other issues that should be considered for assessing the quality of an experimental cell line. By quality, we mean those characteristics that allow assessment of the appropriateness of the cell line for its purpose. As we consider how to develop and encourage the use of methods and standards for qualifying cell lines, we should keep in mind that the use of cell lines in biomedical research is only one application that requires quality assessment. The need for quality assessment of cell lines that are used for clinical products in the form of cell and/or gene therapies and for production of pharmaceutical products will inform and be informed by the research community’s needs for rapid and reliable quality control data. These methods and standards may also find application to identification and quality assurance of biopsied tissues and food products. Similarly, cell lines modified in the context of synthetic biology also require technologies for qualification.

Here we suggest a comprehensive view of cell line assessment in order to lay out a larger landscape that might be considered as the community makes decisions about technologies and standards in this arena.

## Comprehensive Assessment of the Quality of Cell Lines

There are a number of methods available that provide relevant data on the quality of cell lines, and some of the more commonly used methods appear in Table 1. Karyotyping is most often used for detection of gross abnormalities in chromosomes, but chromosome number was the basis of early reports of cell line misidentification [16]. Karyotyping is a specialized technique requiring high technical expertise, time, and significant expense. DNA sequence-based methods such as STR profiling [17,18], single nucleotide polymorphisms (SNP) profiling [19–21], and the use of species-specific primers [22–24] allow detection of mutations, genetic drift, and the presence of adventitious agents [25]. Verification of the species of a cell line can be performed by probing the sequence associated with species-specific cytochrome c oxidase subunit 1 (CO1), commonly referred to as DNA barcoding [26,27], or by employing PCR assays with other species-specific primers [23].

**Table 1:** Some commonly used methods applicable for assessing quality of cell lines.

Quality assurance metric	CO1 Barcode	Karyotype	STR	SNP	Species-specific primers^+^	WGS^++^
Species identification	X	X^%^	X		X	X
Identity of donor individual			X	X		X
Large chromosomal structural changes		X				X
Spontaneous mutations/genetic drift			X^@^	X^@^		X
Contamination with adventitious agents*					X	X
Interspecies contamination	X	X^%^	X^#^	X^#^	X	X
Intraspecies contamination			X	X		X
Phenotypic changes detected with methods other than genomic^**^	Specialized assays					

^**+**^ [22]

^++^ Whole genome sequencing (WGS) is capable of addressing all of these metrics provided there is sufficient bioinformatics support for the interrogation of genomic sequence databases.

^%^ Chromosome number was the basis of early reports of cell line misidentification [16].

^@^ These methods will only be able to detect mutations in the regions of DNA that are covered by the probes or amplicons in the specific assay.

* Other tests (including enzymatic) that are specific for mycoplasma are readily available and are strongly recommended to determine this frequent contamination.

^*#*^ If probes or primers specific for other species are included in the assays.

** Changes due to culture conditions and independent of any genomic changes would have to be carefully controlled for. Quantitative methods for assessing would need to be accompanied by benchmarks that allow comparison of data between laboratories.

Sequence-specific profiles of SNP and STR provide the power to discriminate between individuals of the same species. Interspecies contamination can also be detected with these methods if appropriate primers or probes are included in the test. Whole genome sequencing (WGS) is a powerful technique that, in principle, can provide information about species identity as well as the presence of mutations and contaminating species, including adventitious agents. At the current time, the technical and bioinformatic resources required make WGS expensive; however, as the cost continues to decrease for sequencing and bioinformatic approaches become more routine, this approach will become more practical for routine characterization of cell lines. Phenotypic characteristics such as morphology or gene expression profiles can also be indicative but can be strongly influenced by the culture environment, so strict experimental controls would have to be in place to conclude misidentification. But if one wants to reproduce an experiment, quantitative phenotypic and gene expression profiles can support a claim that cell culture environment is highly similar. Depending on circumstances, it is possible that all of the methods listed in Table 1 would be appropriate for thoroughly assessing a particular cell line. No one method will provide all of the qualifying information that might be desired. As the biomedical community considers how to approach comprehensive cell line qualification, all the methods listed are likely to play a role. Some methods, such as STR profiling and DNA barcoding, are better developed than others at this time, and standards based on them can be developed and put into practice more quickly for qualifying cell lines than can other methods.

The development of community-vetted documentary standards for these methods can play an important role in their acceptance and ease of use. The ANSI/ATCC document on STR profiling for cell line authentication that was developed by an international working group of experts led by ATCC and contributed to by NIST and others is an excellent example of how to develop comprehensive standards. While most if not all of the supporting information for the standard is provided in traditional peer-reviewed publications such as [28], official standards produced under the auspices of ANSI and ANSI-accredited standard development organizations (SDOs) such as ATCC are developed under a formal framework that ensures that appropriate principles and procedures are followed. The formality of the process adds to the credibility of the product and provides confidence to the community that the recommendations are the result of fully expert consideration and consensus. ANSI standards require the participation of all major stakeholders in their development, and comments from the public are generally encouraged. The formality of the process enhances the level of confidence the community has in the resulting document. Because of the adherence to a formal prescribed process, the inclusivity of opinions, the level of knowledge from the contributing experts and the cited literature, and the completeness of the document in terms of rationale, methodologies, and caveats, the ASN-0002 document is a dependable and definitive resource for guiding the community in implementing these methods in a manner in which the results and interpretations from different laboratories can be compared and trusted. The copyright for the standard document is held by ATCC, and the document is readily available for a fee of $240; the fee helps to offset the administrative costs incurred during development and distribution. The standard document resulted from an effort by an international community of 25 experts, who are listed in S1 Text.

## Human Cell Line Authentication: STR Profiling

For the development of ASN-0002, STR profiling was chosen as the preferred method for human cell line authentication because of the extensive work that has been done in human identification for forensics purposes. Forensic identification is almost exclusively based on STR analysis for the following reasons: (1) discriminatory power, (2) established testing infrastructure, (3) cost, (4) effectiveness for degraded DNA samples, (5) comparability of STR profiling data from various platforms, and (6) ability to detect human DNA mixtures [29]. As a result of work in forensics, STR markers that provide good discriminatory value are available for human cell line authentication, and services for paternity and forensics testing are readily adaptable to human cell line authentication.

The ASN-0002 documentary standard is summarized in an electronic book that is available online [30] at http://www.ncbi.nlm.nih.gov/books/NBK144066/. STR profiling was selected as the basis of a standard for human cell line authentication because of its ability to discriminate human cell lines with a relatively small number of allelic markers; comparable data can be achieved across different laboratories, making it feasible to establish a database against which data can be compared; STR profiling kits are available so tests can be run in individual laboratories; it is a relatively rapid and low-cost test; and it doesn’t require as high a level of technical expertise as some other methods.

The standard consists of over 100 pages that include detailed protocols for DNA extraction, STR profiling, data analysis, quality control of the data, interpretation of results, and implementation of a public searchable database. Different methods are compared, all necessary algorithms are provided, and guidance on how to evaluate and interpret data and troubleshoot anomalies is described. How to assess the quality of the data and the interpretation are addressed rigorously, including the use of an allelic ladder (a DNA mixture containing the different alleles permitting calibration), how to assess assay artifacts such as stutter and allelic dropout, appropriate controls, accurate quantification of DNA, etc. A carefully considered basis for assigning a “match” or “mismatch” is based on a matching metric of ≥80% [31]. The careful analysis of what STR typing will provide and what its limitation are vis a vis cell line authentication and the detailed protocols provided make this document a vital resource for the practitioner even beyond its usefulness as a standard. The document is a source of concepts and a thorough reference for good laboratory practice. It is a document that should be familiar to every laboratory performing work with cell lines or providing services. This document also provides a basis for additional standards and refinements that take into account new data and knowledge as they become available. For example, a question regarding differences in the algorithms for determination of matches has recently appeared [32]. The existence of an expert group that is responsible for the formal document provides an excellent forum for discussions and clarifications as needed.

As a result of this standard, the STR profiles of many human cell lines have been collected by the Biological Resource Centers (BRCs), namely the ATCC, the Deutsche Sammlung von Mikroorganismen und Zellkulturen (DSMZ), the Japanese Collection of Research Bioresources (JCRB), and RIKEN, and these data are available in public databases (http://www.atcc.org/STR%20Database.aspx; http://www.jhsf.or.jp/bank/Category-Index.html; https://www.dsmz.de/services/services-human-and-animal-cell-lines/online-str-analysis.html). The DSMZ database allows profile searching against cell lines from all of these databases. The existence of STR profile databases is critical for providing a benchmark profile against which a user can compare their cell line by searching by name of the cell line or by STR profile. The National Center for Biotechnology Information (NCBI) also hosts the BioSample database (http://www.ncbi.nlm.nih.gov/biosample/), which contains the STR profiles of the most frequently used cell lines from the BRCs (listed above) as well as the International Cell Line Authentication Committee *(*ICLAC) database (http://iclac.org/databases/cross-contaminations/), which list human cell lines that are misidentified based on their STR profiles.

At this time, there is no definitive determination regarding how often authentication and contamination testing should be performed, although a number of publications have addressed this issue [1]. As more testing is done, collection and consolidation of supporting data may provide evidence on which recommendations or standards could be based.

## Alternative Methods for Human and Nonhuman Cell Line Authentication

While STR profiling is the only method that is part of a formal consensus standard for distinguishing cell lines from the same species, this standard pertains only to human cell lines. Table 2 compares the state of development of the common methods used to establish the identity of cell lines for human and non-human species.

**Table 2:** Current status of SNP, STR, and DNA barcode technologies as standard methods for assessing the identity of cell lines from different species.

**Species**	**Assays**	**Consensus Standard Method**	**Commercially Available Kit**	**Commercial Service**	**Comparative Data**
**Human**	STR	ASN-0002	Yes	Yes	ATCC, DSMZ, JCRB, NCBI**
	SNP	No	Yes	Yes	[21], [32], NCBI
**Mouse**	STR*	No	No	Yes	Unpublished
	SNP	No	Yes	Yes	[19]
**African green monkey**	STR*	No	No	No	None
**Chinese hamster ovary**	STR*	No	No	No	None
**Rat**	STR*	No	No	No	None
**Species-level identification**	CO1 DNA barcode	ASN-0003	Yes	Yes	Barcode of Life Data System, NCBI**
	Species-specific primers	No	No	Yes	None needed

These methods are currently the most developed for this application. There are extensive data on human cell lines, but while there are some kits and services for some nonhuman species, there is little available data for nonhuman species, except for DNA barcoding, which only distinguishes cell lines on the basis of species, not individuals.

* STR markers have been identified [33,34]. Markers for rat and Chinese hamster ovary cells are still under development by NIST.

** These sources contain a significant amount of data from multiple sources. See text for URLs.

Standards for using SNP markers for authentication of human cell lines have been proposed [20,21] and some data have been collected (http://www.ncbi.nlm.nih.gov/snp). Standard methods for CO1 DNA barcoding for distinguishing animal cell lines at the level of species have been developed by the Consortium for the Barcode of Life [35]. The ATCC/ANSI standard (ASN-0003) for DNA barcoding entitled *Species-Level Identification of Animal Cells through Mitochondrial Cytochrome c Oxidase Subunit 1 (CO1) DNA Barcodes* is now available [36]. The contributors to this standard are listed in S2 Text. DNA barcoding is extremely useful in determining the species of a cell line by targeting the CO1 gene and searching the databases (BOLD, http://www.barcodinglife.org, and NCBI, http://www.ncbi.nlm.nih.gov/genbank/barcode) for matches. Species-specific primer assays [22,23] provide a quick and inexpensive way for a lab to confirm the species identity of a sample by running a multiplex PCR assay and separating the amplicons by fragment length. The only species that will be detected are those that are represented in the assay. Not all animal species are included in the assays. A consensus standard for the use of species-specific multiplex PCR is being organized by ANSI/ATCC. Species-specific primers have the advantages that they use low-cost instrumentation and the analysis is straightforward, but they do not allow cell line identity testing or detection of intraspecies contamination.

## Authentication of Nonhuman Cell Lines

When considering authentication of nonhuman cell lines, the extensive body of technical knowledge on STR profiling, which is captured in the ANSI standard and by other organizations (e.g., The International Society for Forensic Genetics), and the proven record of practicality and fit-for-purpose of STR profiling, make it an obvious choice for consideration as a standard method for nonhuman cell lines.

A number of issues that accompanied the development of the ANSI/ATCC standard for human cell lines will have to be considered for nonhuman cell lines. These include the following: identifying reliable polymorphic STR markers that provide intraspecies discrimination; having many laboratories collect STR data on the same cell lines, establishing and accumulating consensus data for informative STR markers that produce reliable results to consistently characterize a cell line; determining the metrics by which the raw data will be deemed to be of sufficient quality; and determining the best methods for assessing concordance in STR marker data and what level of concordance is sufficient to call a “match.” These are tasks that will need to be undertaken in a community effort. STR databases for nonhuman species need to be developed using this process.

Having community-vetted documentary standards is a critical factor that is helpful for facilitating implementation of cell line authentication. Table 2 summarizes the progress so far on the tools that are beginning to appear for authentication of human and most commonly used nonhuman cell lines and indicates the existing gaps. For mouse cell lines, SNP [19] and STR [33] markers have been identified, and STR markers have been identified for African green monkey [34]; markers are under development for Chinese hamster ovary (CHO) and rat cell lines. However, comprehensive testing of existing markers for their discriminatory power and databases for different cell lines against which researchers can compare their cell lines need to be developed. As authentication becomes common practice in more laboratories, it will likely be made easier, because more companies will provide the needed services. Only a small number of companies currently provide STR profiling services for mouse cell lines. To spur the development and availability of services, a consortium of organizations and companies is being planned to create an STR marker kit and compile data on STR profiles of mouse cell lines to develop a public database.

## Conclusions

Measurements can provide confidence in the use of cell lines. Community action around how to apply measurements reliably and the development of comparative data are key to achieving a comprehensive toolkit for assuring the quality of cell lines. The existence of the ANSI/ATCC standard (ASN-0002) for STR profiling of human cell lines and ASN-0003 for species barcoding are critical first steps and provide excellent examples that can be built on. Similar activities should be undertaken for the other methods discussed here.

Despite the existence of the very thorough and highly vetted documentary standard, human cell line authentication is still not commonly performed. In part, this may be due to the lack of awareness of this document and the need for increased education and training programs on cell line authentication. The ASN-0002 document could provide an excellent basis for educational materials to be used for training in procedures for achieving robust qualifying data. It is also likely that greater availability of contract testing services and clearer funding and publishing priorities will be required to stimulate the application of the capabilities and standards that are already in place for human cell line identification and to spur the development of similar standards for the important nonhuman cell lines. Consensus standards that are produced in a careful, open, and official process are an integral part of the success of this endeavor. Standards help to assure that data are sharable and can be the basis of decision-making and compliance. The collection of high-quality data from a large range of cell lines and different labs is an initial necessary step. What will follow are consensus standards and access to the data, which will facilitate labs and companies to provide the testing services and encourage the widespread adoption of this essential part of quality assurance of any process that utilizes cell lines.

## Supporting Information

S1 Text Participants in the Working Group for ASN-0002, *Authentication of Human Cell Lines*: *Standardization of STR Profiling*. (DOCX)

S2 Text Participants in the Working Group for ASN-0003, *Species-Level Identification of Animal Cells through Mitochondrial Cytochrome c Oxidase Subunit 1 (CO1) DNA Barcodes*. (DOCX)

## Abbreviations

ANSI: American National Standards Institute
ATCC: American Type Culture Collection
BRC: Biological Resource Center
CHO: Chinese hamster ovary
CO1: cytochrome c oxidase subunit 1
DSMZ: Deutsche Sammlung von Mikroorganismen und Zellkulturen
ICLAC: International Cell Line Authentication Committee
JCRB: Japanese Collection of Research Bioresources
NIH: National Institutes of Health
SDO: standard development organization
SNP: single nucleotide polymorphism
STR: short tandem repeat
WGS: whole genome sequencing
