# Supplementary material for: Standards for Cell Line Authentication and Beyond
Source: PLoS Biol. 2016 Jun 14;14(6):e1002476. doi: 10.1371/journal.pbio.1002476 (PMC4907466; doi:10.1371/journal.pbio.1002476)
Supplement: S1 Text — (DOCX) [file pbio.1002476.s001.docx]

| **John R. W. Masters, PhD, FCR Path**  [Workgroup Chair]  Professor of Experimental Pathology  Division of Surgery and Interventional Sciences  University College London  67 Riding House Street  London W1W 7EJ UK | **Arihiro Kohara, PhD**  Scientist  National Institute of Biomedical Innovation  Department Biomedical Resources,  Laboratory of Cell Cultures  7-6-8 Saito-Asagi, Ibaraki  Osaka, Japan 567-0085 |
| --- | --- |
| **Yvonne A. Reid, PhD**  **[***Workgroup Co-Chair*]  Manager, Scientist  Cell Biology Program  American Type Culture Collection  10801 University Blvd.  Manassas, VA 20110 | **Roland M. Nardone, PhD**  Professor Emeritus  Catholic University of America  Cell and Molecular Biology  620 Michigan Ave. NE  Washington, DC 20064 |
| ***Workgroup members:***  **Raymond W. Nims, PhD**  **[**Rationale Subgroup *Chair*]  Consultant  RMC Pharmaceutical Solutions, Inc.  2150 Miller Drive, Suite A  Longmont, CO 80501 | **Yvonne A. Reid, PhD**  [*Protocol Development & Database Subgroup Chair*]  Manager, Scientist  Cell Biology Program  American Type Culture Collection  10801 University Blvd.  Manassas, VA 20110 |
| **Steven R. Bauer, PhD**  FDA/Center for Biologics Evaluation and Research  Chief, Cell and Tissue Therapy Branch  Division of Cellular and Gene Therapies  Office of Cellular, Tissue, and Gene Therapies  8800 Rockville Pike  Bethesda, MD 20892 | **Georgyi V. Los, MD, PhD**  **[***Protocol Development & Database Subgroup Secretary*]  Life Science Research  Thermo Fisher Scientific  100 Technology Drive  Pittsburgh, PA 15219 |
| **Manohar Furtado, PhD**  VP R & D,  Applied Markets Division  Applied Biosystems  850 Lincoln Centre Drive, MS404-1  Foster City, CA 94404 | **Wilhelm G. Dirks, PhD**  Molecular Biologist  DSMZ, Deutsche Sammlung für Mikrorganismen und Zellkulturen  Inhoffenstr. 7b  38124 Braunschweig  Germany |
| **Margaret C. Kline**  Research Biologist  Biochemical Science Division (831)  Advanced Chemical Science Laboratory (227),  Room B226  NIST  100 Bureau Drive, Stop 8311  Gaithersburg, MD 20899-8311 | **Jaiprakash G. Shewale, PhD**  Technical Director, HID Genetic Systems  Life Technologies  850 Lincoln Centre Drive  Foster City, CA 94404 |

| **Paul J. Price, PhD**  Consultant  Cellomics Life Science Research  ThermoFisher Scientific  100 Technology Drive  Pittsburgh, PA 15219 | **Anton F. Steuer, PhD**  Program Management Technical Director  BioReliance Corporation  14920 Broschart Road,  Rockville, MD 20850 |
| --- | --- |
| **Jim Thomson, PhD**  Innovation and Support Team  LGC  Queens Rd, Teddington, TW11 0LY, UK | **Douglas R. Storts, PhD**  Head of Research  Nucleic Acid Technologies  Promega Corporation  2800 Woods Hollow Road  Madison, WI 53711 |
| ***Alternate members:***  **Amanda Capes-Davis, MB BS BSc(Med) PhD**  Founding Manager, CellBank Australia  Children‟s Medical Research Institute  214 Hawkesbury Road,  Westmead NSW 2145 Australia | **Gregory Sykes**  Biologist  Molecular Authentication Resource Center  American Type Culture Collection  10801 University Blvd.  Manassas, VA 20110 |
| **Rita Barallon, PhD**  Service Business Manager- Life and Food Sciences  Life Sciences  LGC  Queens Road, Teddington, Middlesex, TW11 0LY | **Zenobia F. Taraporewala, PhD**  FDA/Center for Biologics Evaluation  and Research Reviewer Division of Cellular and Gene Therapies Office of Cellular, Tissue, and Gene Therapies  1401 Rockville Pike, Room 200N  Rockville, MD 20852 |
| **John M.Butler, PhD**  Biochemical Science Division (831)  Advanced Chemical Science Laboratory (227), Room B250  NIST  100 Bureau Drive, Stop 8311  Gaithersburg, MD 20899-8311 | ***ATCC Workgroup Support Personnel:***  **Liz Kerrigan**  Director, ATCC SDO  American Type Culture Collection  10801 University Blvd.  Manassas, VA 20110-2209 |
| **Eugene Elmore, PhD**  Department of Radiation Oncology  University of California  Medical Sciences I, B146D  Irvine, CA 92697 | **Christine Y. Alston-Roberts**  Standards Specialist  American Type Culture Collection  10801 University Boulevard  Manassas, VA 20110-2209 |
| **Roderick A. F. MacLeod, PhD**  Cytogenetist  DSMZ, Deutsche Sammlung für Mikrorganismen und Zellkulturen  Inhoffenstr. 7b  38124 Braunschweig  Germany | |
