# Supplementary material for: Standards for Cell Line Authentication and Beyond
Source: PLoS Biol. 2016 Jun 14;14(6):e1002476. doi: 10.1371/journal.pbio.1002476 (PMC4907466; doi:10.1371/journal.pbio.1002476)
Supplement: S2 Text — (DOCX) [file pbio.1002476.s002.docx]

| Jason Katz Cooper, MS [Workgroup Chair]  Biology Professor  Community College of Beaver County  1 Campus Drive, Monaca, PA 15061  Yvonne Reid, PhD [Workgroup Co-Chair]  Manager/Scientist, Cell Biology Program  ATCC  10801 University Boulevard, Manassas, VA 20110  **Workgroup members**:  Christopher Korch, PhD [Sub-group Chair]  Assistant Clinical Professor  Division of Medical Oncology  Campus Mailstop 8117  University of Colorado School of Medicine  12801 East 17th Avenue  Aurora, CO 80045  Nancee Oien [Sub-group Chair]  Associate Research Fellow  VMRD Biologicals Development, Analytical  Zoetis  333 Portage Road, Kalamazoo, MI 49007  Gregory Sykes, MS [Sub-group Chair]  Biologist  ATCC  10801 University Boulevard  Manassas, VA 20110-2209  Jamie Almeida, MS  Microbiologist  Bioassay Methods Group,  Biochemical Science Division  National Institute of Standards and Technology (NIST)  100 Bureau Drive, Mailstop 8312  Gaithersburg, MD 20899-8312  Beth Bauer, DVM  Head of IDEXX BioResearch  Genetic Services  4011 Discovery Drive  Columbia, MO 65201  Edward Burnett, PhD  Scientific Development Group Leader,  Culture Collections  Public Health England (PHE)  Manor Farm Road  Main Building, Room 01/212-1  Wiltshire, Salisbury  England SP4 0JG  Afshin Sohrabi, PhD  Senior Scientist  Molecular Development Services  BioReliance by SAFC  14920 Broschart Road  Rockville, MD 20850  Mark Stoeckle, MD  Senior Research Associate  Program for the Human Environment  Nurses Residence, Room A411  The Rockefeller University  1230 York Ave  New York, NY 10065 | Kenneth Cole, PhD  Team Leader  Bioassay Methods Group  National Institute of Standards and Technology (NIST)  100 Bureau Drive, Mailstop 8312  Gaithersburg, MD 20899-8312  James (Jim) Cooper, PhD  Cell Biology Applications Scientist,  Culture Collection  Public Health England (PHE)  HPA Portion  Salisbury, Wiltshire  United Kingdom SP4 0JG  Manohar Furtado, PhD  Senior Director, Technical Compliance  Cepheid, Sunnyvale CA  Bob Hanner, PhD  Associate Professor  Biodiversity Institute of Ontario &  Centre for Biodiversity Genomics  University of Guelph  50 Stone Road East  Guelph, ON  N1G 2W1  Canada  Arihiro Kohara, PhD  Manager, Scientist  National Institute of Biomedical Innovation  7-6-8, Saito-Asagi, Ibaraki  Osaka, Japan 567-0085  Ray Nims, PhD  Senior Consultant  RMC Pharmaceutical Solutions, Inc.  Suite A, 1851 Lefthand Circle  Longmont, CO 80501   \| David E. Schindel, PhD  National Museum of Natural History  Smithsonian Institution  P.O. Box 37012, MRC-105  Washington, DC 20013-7012  Silke Schlottmann, PhD  Division of Microbiology Devices  Office of In Vitro Diagnostics and Radiological Health (OIR)  Center for Devices and Radiological Health  U.S. Food and Drug Administration  10903 New Hampshire Avenue  Building 66, Room 4566  Silver Spring, MD 20993-0002 \| \| --- \| |
| --- | --- | --- |
